# Effect of Ni/Si Mass Ratio and Thermomechanical Treatment on the Microstructure and Properties of Cu-Ni-Si Alloys

**DOI:** 10.3390/ma12132076

**Published:** 2019-06-27

**Authors:** Jiang Li, Guojie Huang, Xujun Mi, Lijun Peng, Haofeng Xie, Yonglin Kang

**Affiliations:** 1 State Key laboratory of Nonferrous Metals and Processes, GRIMAT Engineering Institute Co., Ltd., Beijing 101407, China; 2 School of Materials Science and Engineering, University of Science & Technology Beijing, Beijing 100083, China

**Keywords:** Cu-Ni-Si alloy, Ni/Si mass ratio, physical properties, microstructure, thermomechanical treatment, theoretical calculation

## Abstract

The effect of the Ni/Si mass ratio and combined thermomechanical treatment on the microstructure and properties of ternary Cu-Ni-Si alloys is discussed systematically. The Cu-Ni-Si alloy with a Ni/Si mass ratio of 4–5 showed good comprehensive properties. Precipitates with disc-like shapes were confirmed as the Ni_2_Si phase with orthorhombic structure through transmission electron microscopy, high-resolution transmission electron microscopy, and 3D atom probe characterization. After the appropriate thermomechanical treatment, the studied alloy with a Ni/Si mass ratio of 4.2 exhibited excellent mechanical properties: a hardness of 290 HV, tensile strength of 855 MPa, yield strength of 782 MPa, and elongation of 4.5%. Compared with other approaches, the thermomechanical treatment increased the hardness and strength without sacrificing electrical conductivity. Theoretical calculations indicated that the high strength was primarily attributed to the Orowan precipitation strengthening and secondarily ascribed to the work hardening, which were highly consistent with the experimental results. The appropriate Ni/Si mass ratio with a low content of Ni and Si atoms shows high strength and excellent electrical conductivity through combined thermomechanical treatment. This work provides a guideline for the design and preparation of multicomponent Cu-Ni-Si-X alloys with ultrahigh strength and excellent electrical conductivity.

## 1. Introduction

Cu-Ni-Si system alloys have wide electrical applications, such as in circuits, connectors, and lead frames, where high mechanical properties combined with excellent electric conductivity and thermal conductivity are required [1,2]. In the last decades, outstanding comprehensive properties have been achieved for age-hardening alloys. This accomplishment contributes to the detailed understanding of the uniform distribution of nano-scale Ni-Si precipitates and helps in determining the properties of the matrix. For instance, some alloys with low Ni and Si content, such as KLF-1, C70250, and C70350, have favorable electrical conductivity (>45% IACS) and are used for high-density miniaturized electronic components. Others with high content of Ni and Si have superior strength (>1000 MPa) and micro-hardness (>300 HV), which are advantageous in the field of structural materials [3,4].

Studies on Cu-Ni-Si system alloys have mainly focused on three aspects: first is the effect of Ni/Si ratio on the microstructure and properties of Cu-Ni-Si alloys, second is the different heat treatments for alloys that were explored to enhance their physical properties, and the last is the addition of microelements to Cu-Ni-Si alloys to synthesize a new multi-element alloy with optimal comprehensive performance. To date, most works have focused on the latter two. The ratio of Ni and Si should also be considered as the basis of the chemical composition designation for Cu-Ni-Si alloys. Lockyer [5,6] first proposed that the amount of Ni and Si greatly affects the properties of Cu-Ni-Si alloys. Fujiwara [7] and Semboshi [8] provided a preliminary reference for selecting the appropriate Ni/Si ratio with low addition of Ni and Si. However, the relationship between the Ni/Si ratio and microstructure cannot be observed during aging. Lei [9] also studied the microstructure evolution of Cu-Ni-Si alloys with high Ni and Si content and found that the microstructures of Cu-Ni-Si alloys are attributed to the change in the number of δ-Ni_2_Si phases formed within the grains and the transition from Cu-Si to Ni-Si phase in the grain boundary. With the increase in Ni/Si atomic ratio from 0.33 to 3, the precipitated phase changes from Cu_5_Si to Ni_3_Si phase. According to these studies, the relationship between microstructure (grain size and precipitation characteristics) and the properties of Cu-Ni-Si alloys with different Ni/Si mass ratios during aging is poorly understood and characterized. In general, the chemical composition designations of Cu-Ni-Si alloys require suitable Ni/Si mass ratios that can stabilize all relevant phases and reproduce their optimal properties with reasonable accuracy.

Some controversies exist about the precipitation characteristics of phases in Cu-Ni-Si system alloys. Corson [10] first presented the precipitated phase as the δ-Ni_2_Si phase based on the Cu-Ni-Si ternary phase diagram. Lei [11] pointed out that the δ-Ni_2_Si phase is formed in the peak aging state, and the precipitation of the β-Ni_3_Si phase occurs throughout the aging process. Chen [12] identified that the micro-shape characteristics of the β-Ni_3_Si phase evolve from spheroid to ellipsoid, whereas δ-Ni_2_Si maintains its disk shape during precipitation. However, Jia [13] revealed six variants of δ-Ni_2_Si phases and found that the β-Ni_3_Si phase cannot be found in the alloy. Hu [14] further reported that the second phase is the δ-Ni_2_Si phase and the crystallographic structure evolves during aging. According to the principle of minimum energy, the δ-Ni_2_Si phase changes from an almond-like δ_1_ to a bread-slice-like δ_2_ precipitate. Our team [15] also conducted a preliminary exploration on the microstructure of the Cu-Ni-Co-Si alloy and confirmed that the phase is (Ni, Co)_2_Si. To date, the controversies about the scientific evidence for the crystallographic structure and morphology of precipitates in the Cu-Ni-Si system alloys have remained unabated. The effect of the precipitation characteristics of the ternary Cu-Ni-Si alloy has not been deeply and systematically studied and therefore has become the focus of this paper.

In this study, the ternary Cu-Ni-Si alloy with different Ni/Si mass ratios was systematically designed. The relationship between the properties and microstructure of Cu-Ni-Si alloys with different Ni/Si mass ratios is discussed. Moreover, the morphology and orientation relationship (OR) of precipitates in the Cu-Ni-Si alloy were characterized in detail using a transmission electron microscope (TEM) and a high-resolution transmission electron microscope (HRTEM). The precipitation type of the second phases and their atomic distribution in the studied alloy were investigated using the 3D atom probe (3DAP) technique. Different heat treatments were applied to identify the outstanding comprehensive properties and the appropriate Ni/Si mass ratio.

## 2. Experimental Procedures

The compositions of the Cu-Ni-Si alloys are shown in Table 1. Ingots of studied alloys were melted in an intermediate frequency furnace. After surface defects were removed, the cast ingots were hot rolled to 2-mm thickness at 930 °C and cut into sheets with the dimensions of 25 × 20 × 2 mm^3^. A subsequent solution treatment was performed for 1 h at 900 °C, followed by quenching in water. The sheets were treated by either single-step or combined heat treatment. The former underwent isothermal aging at 450 °C and 500 °C for various times, whereas the latter underwent treatments in different conditions as presented in Table 2.

Vickers hardness was examined through indentation test on a WILSON VH1150 testing machine (Chicago, IL, USA) at a load of 5 kg and a holding time of 15 s. Electrical conductivity was analyzed using a Sigma 2008 (St. Louis, MI, USA) digital eddy current conductivity meter. Each measured value was calculated from the average of 10 data points. Tensile specimens were prepared from the peak-aging sheets with an original scale of 40 mm and then examined on an MTS-WD 3100 instrument (Eden Prairie, MN USA) with a constant rate of 3 mm/min. Metallographic observation was performed using a type of Axiovert 200MAT light microscope (Zeiss, Jena, Germany). Thin foils for TEM observation were prepared by double-jet electrolytic polishing in a solution of 25% nitric acid and 75% methanol below −40 °C. TEM and HRTEM were conducted under a FEI Tecnai G^2^ F20 (Hillsboro, OR, USA) electron microscope with an operating voltage of 200 kV. The 3DAP experiments were performed on a LEAP 4000 HR instrument (Cameca, Gennevilliers, France) with a temperature of 50 K and a pulse rate of 200 kHz. The samples for 3DAP analysis were cut into needle-like shape with a size of 0.5 × 0.5 × 20 mm^3^, followed by electro-polishing according to the standard two-stage methods [16].

## 3. Results

### 3.1. Physical Properties

**Figure 1:**
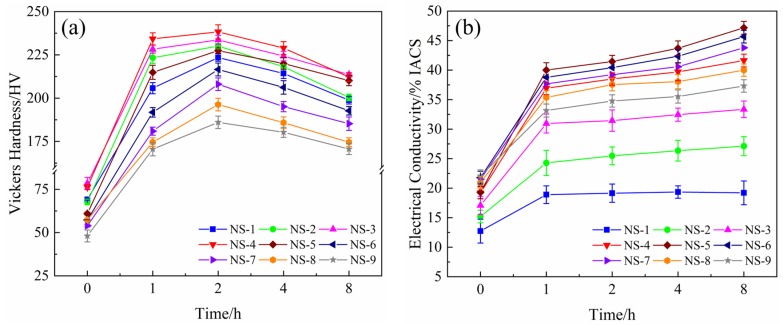
Hardness and electrical conductivity of alloys with different Ni/Si mass ratios. (**a**) Hardness and (**b**) electrical conductivity.

Figure 1 shows the hardness and electrical conductivity curves of Cu-Ni-Si alloys aged at 500 °C for different times. As presented in Figure 1a, the hardness of the specimens increases rapidly, reaches the peak value, and then decreases during aging. For the different Ni/Si mass ratios of alloys, the hardness shows the same tendency in the whole time period, and the peak aging is generally achieved under the aging condition of 500 °C for 2 h. With regard to the hardness, the relationship between electrical conductivity and aging time in the Cu-Ni-Si alloys with different Ni/Si mass ratios presents the same tendency as shown in Figure 1b. With the increase in aging time, the electrical conductivity of specimens also increases rapidly in the initial aging, then increases slowly, and finally remains steady in the end.

**Figure 2:**
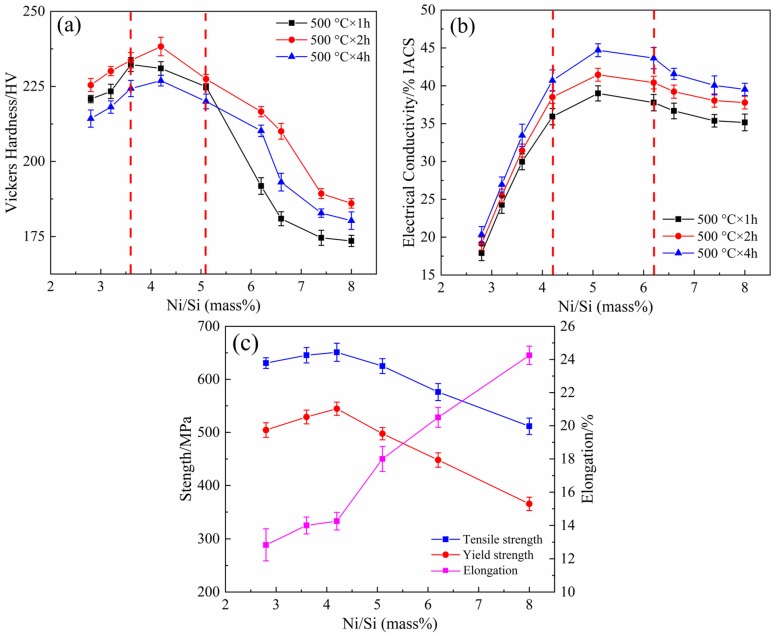
Mechanical properties and electrical conductivity of alloys with different Ni/Si mass ratios. (**a**) Hardness, (**b**) electrical conductivity and (**c**) tensile properties.

Figure 2 illustrates the variations of mechanical properties and electrical conductivity of alloys aged at 500 °C with different Ni/Si mass ratios. Under the same aging conditions, the hardness increases to the peak and then drops sharply with the increase in Ni/Si mass ratios as shown in Figure 2a. The hardness is high when the Ni/Si mass ratio is 3.6–5.1 (NS-2-NS-5) and reaches the maximum value when the Ni/Si ratio is 4.2 (NS-4). For example, the peak value of micro-hardness is 238 HV when the alloy is aged at 500 °C for 2 h. The influence of different Ni/Si mass ratios on the conductivity of alloys is shown in Figure 2b. This result indicates that the conductivity is good when the Ni/Si ratio is at 4.2–6.2 (NS-4-NS-6) and reaches the peak at a 5.1 ratio (NS-5). Considering the combination of hardness and electrical conductivity, the Ni/Si mass ratio of 4–5 (NS-4) provides good properties, and the peak aging system is at 500 °C for 2 h.

The strength and elongation of alloys with different Ni/Si mass ratios in the peak aging state are presented in Figure 2c. The tensile and yield strength gradually increases at first and then rapidly decreases after reaching the peak value. On the contrary, the elongations gradually improve with the increase in Ni/Si mass ratios. At high Ni/Si ratio ranges, the elongations notably increase, whereas the strength remarkably decreases. The peak values are obtained when the Ni/Si ratio is 4.2 (NS-4), and the corresponding yield strength, tensile strength, and elongation are 544 MPa, 651 MPa, and 14.3%, respectively.

**Figure 3:**
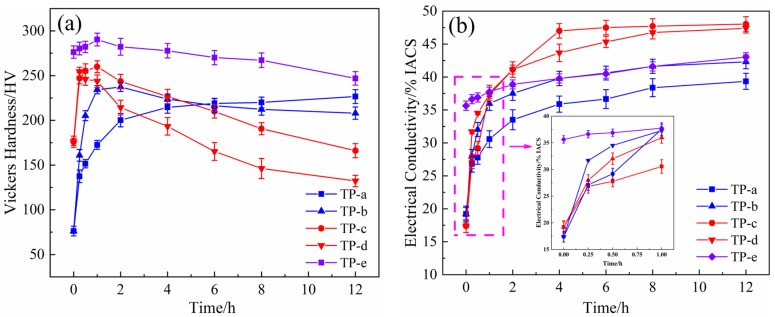
Hardness and electrical conductivity of the NS-4 alloy with different combined aging treatments. (**a**) Hardness and (**b**) electrical conductivity.

Figure 3 exhibits the hardness and electrical conductivity of the NS-4 alloy with different combined heat treatments. The hardness and electrical conductivity of specimens aged at 500 °C (TP-b) are higher than those of specimens aged at 450 °C (TP-a). After aging at 500 °C for 2 h, the specimens exhibit a peak hardness of 238 HV and corresponding electrical conductivity of 37.5% IACS. However, the properties of specimens aged at 500 °C (TP-d) are far inferior to those of specimens aged at 450 °C (TP-c) after the cold rolling as shown in Figure 3a. This finding reveals that over-aging occurs at 500 °C, and the rolling process has declined the peak aging temperature of the alloy. Compared with the specimen at the direct aging state (TP-a, b), the re-aging specimen that underwent cold rolling (TP-c, d) shows better electrical conductivity properties, but its hardness sharply decreases in the late aging period. Therefore, the second cold rolling and aging processes were added to obtain an excellent and stable performance. Sample e (TP-e) underwent first cold rolling, first aging, second cold rolling, and second aging. Its hardness significantly increases, but its conductivity slightly decreases as presented in Figure 3. After aging at 350 °C for 1 h, the NS-4 alloy shows peak hardness at 290 HV and a corresponding electrical conductivity of 37.5% IACS. Compared with those of the TP-b and TP-c, the peak hardness of the NS-4 alloy is remarkably increased by 22% and 12%, respectively.

**Figure 4:**
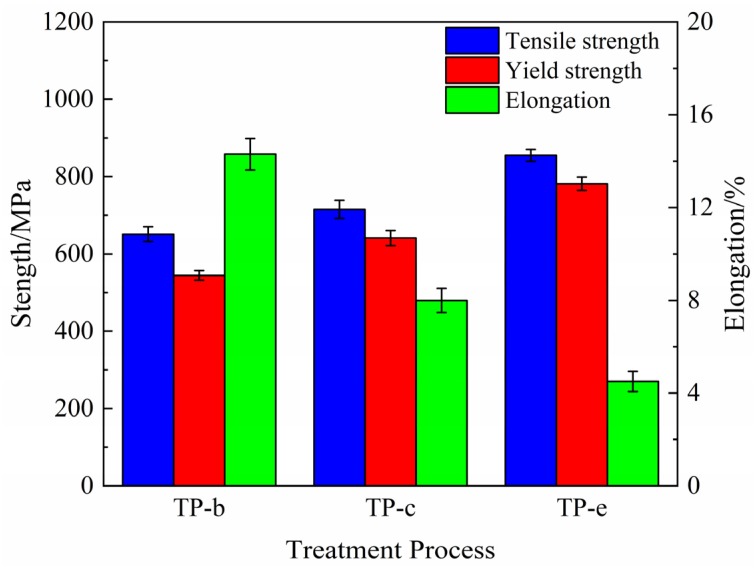
Tensile and yield strength of the NS-4 alloy with different combined aging treatments.

The comparison of results from tensile tests of the NS-4 alloy under peak aging with different heat treatments are shown in Figure 4. The strength of the alloy remarkably improves after the second cold rolling and aging. Compared with those of the TP-b, the tensile and yield strength of the TP-e reach 855 MPa and 782 MPa at 31% and 44% increases, respectively. These results are ascribed to the increase in dislocation density and the acceleration of the nucleation rate of precipitates by the cold rolling, thus resulting in the enhanced strength.

### 3.2. Microstructure Observation

**Figure 5:**
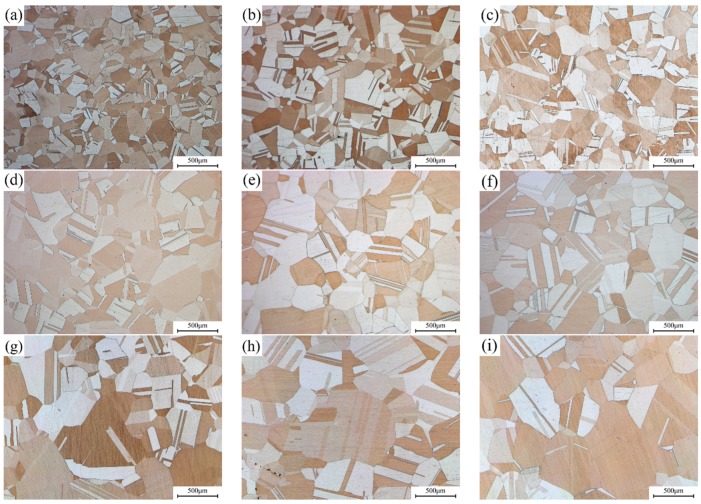
Light microscope images of alloy microstructure with different Ni/Si mass ratios after solution treatment; (**a**–**i**) NS-1–NS-9.

The typical microstructure of Cu-Ni-Si alloys with different Ni/Si mass ratios that underwent solution treatment is shown in Figure 5. The equiaxed grains with twins and no inter-metallic phase are found in the matrix. This finding suggests that Ni and Si atoms are almost dissolved to form a supersaturated solid solution during the solution treatment, thus resulting in the formation of superfluous vacancies in the interior of the matrix and facilitating the precipitation of Ni-Si phases. In addition, the average grain size continues to increase with the increase in Ni/Si mass ratios.

**Figure 6:**
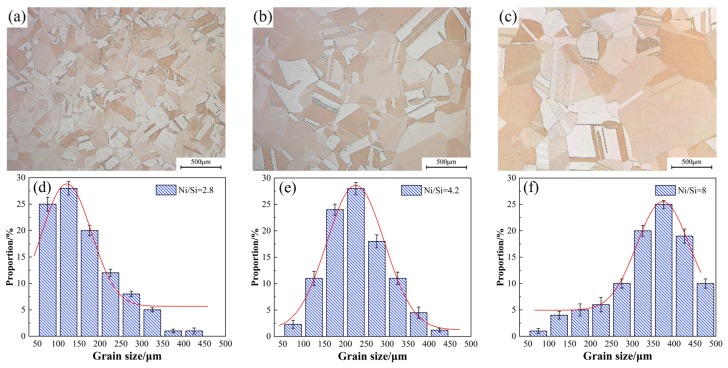
Average grain size of alloys with different Ni/Si mass ratios aged at 500 °C for 2 h; (**a**,**d**) NS-1, (**b**,**e**) NS-4, and (**c**,**f**) NS-9.

Figure 6 presents the light microscope images of alloy microstructure with different Ni/Si mass ratios at the peak aging state. The precipitated particles could not be observed in the micrograph image mainly due to their small size. Other methods were used for further observation. In addition, the average grain size also gradually increases under the aging state, which is consistent with the solution treatment. The statistics of average grain size under the peak aging state in Figure 6 basically presents the function of normal distributions. When the Ni/Si mass ratio increases, the average grain size of the specimen also increases from approximately 145 μm to 350 μm and is accompanied by the growth of the number of twin bands.

**Figure 7:**
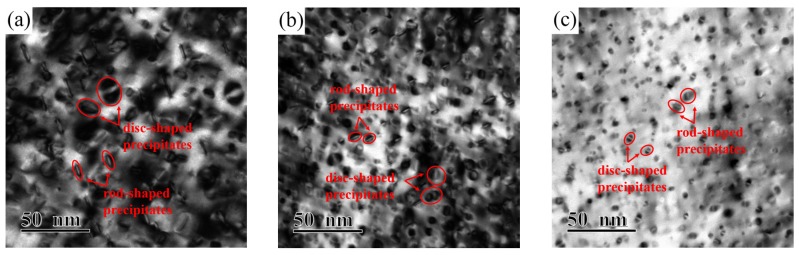

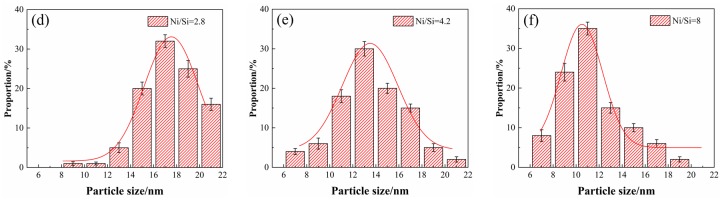
Transmission electron microscopy (TEM) images of alloy microstructure and particle size of alloys with different Ni/Si mass ratios aged at 500 °C for 2 h; (**a**,**d**) NS-1, (**b**,**e**) NS-4, and (**c**,**f**) NS-9.

Figure 7 illustrates the TEM images of alloy microstructure and the particle size distribution of alloys versus different Ni/Si mass ratios along the zone axis of [110]_Cu_. A large number of precipitates with disc- and rod-like shapes had formed and are shown in each bright field image. With the increase in the Ni/Si ratio, uniform and fine precipitates are obtained due to the strengthening of the aged alloy. Compared with that of the NS-1 alloy, the average size of the NS-9 alloy decrease doubled, and the particles are approximately 10–11 nm in size. Finely dispersed phase particles are well distributed in the matrix, and the movement of dislocations and grain boundaries are inhibited. This phenomenon substantially contributes to the improvement of mechanical properties.

**Figure 8:**
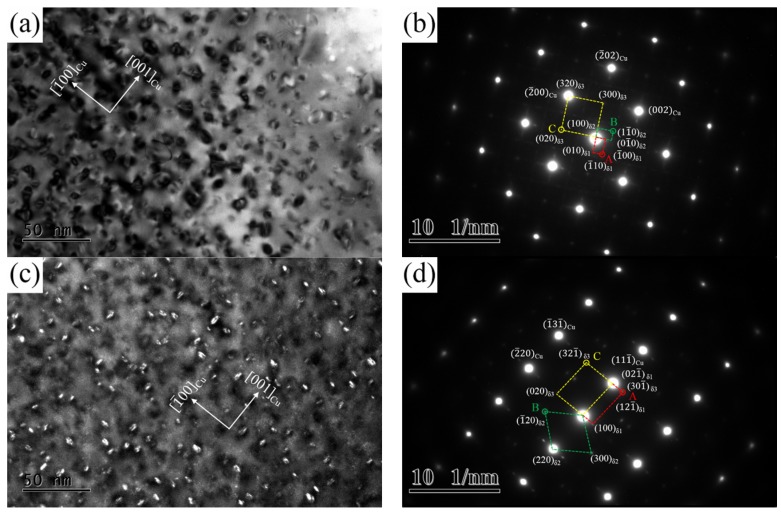
TEM images of the NS-4 alloy aged at 500 °C for 2 h. (**a**) Bright-field TEM image along [001]_Cu_, (**b**) SAED corresponding of (**a**), (**c**) dark-field TEM image of A, and (**d**) SAED corresponding along [112]_Cu_.

The crystallographic and morphological features of precipitates are revealed for the Cu-Ni-Si alloy aged at 500 °C for various times. Figure 8 illustrates the TEM images obtained from the NS-4 alloy in the peak aging state. A large number of bean-shaped precipitation particles are distributed homogenously in the matrix as shown in Figure 8a. The average size of particles is approximately 13–15 nm, which is highly coherent with the copper matrix. As indicated by the corresponding selected-area electron diffraction (SAED) pattern image in Figure 8b, three sets of spots are visible near the matrix spots and are marked as A, B, and C. Spots A and B are collinear and perpendicular to each other along the <100>_Cu_ direction, whereas spot C is distributed in the middle of the ($\bar{2}00$)_Cu_ position [17]. The corresponding OR of precipitates and matrix were measured as follows: ($\bar{2}02$)_Cu_//($0\bar{1}0$)_δ1_//(100)_δ2_//(300)_δ3_, [001]_Cu_//[100]_δ1_//[100]_δ2_//[100]_δ3_. According to the central dark-field image in Figure 8c, the precipitates have a mutually perpendicular relationship, similar crystal lattice, and ORs with the matrix. The existence of three ordering phases and ORs can be further confirmed through calibration along the [112]_Cu_ direction in Figure 8d, the results of which coincide well with the analysis in Figure 8b. Therefore, according to the crystallographic feature of ordering phases in the Cu-Ni-Si alloy, these diffraction spots are identified as δ-Ni_2_Si phases with orthorhombic structure on the basis of the lattice constant (a = 0.706 nm, b = 0.499 nm, c = 0.372 nm) [5,6]. In addition, the Ni_2_Si phase has the lowest formation enthalpy among the different Ni-Si phases, thereby further confirming that the Ni_2_Si phase is the main precipitated phase [18]. The corresponding crystal OR of precipitates and matrix were calibrated as follows: ($11\bar{1}$)_Cu_//($02\bar{1}$)_δ1_//($\bar{2}\bar{2}0$)_δ2_//($30\bar{1}$)_δ3_, [112]_Cu_//[0$\bar{1}\bar{2}$]_δ1_//[001]_δ2_//[103]_δ3_.

**Figure 9:**
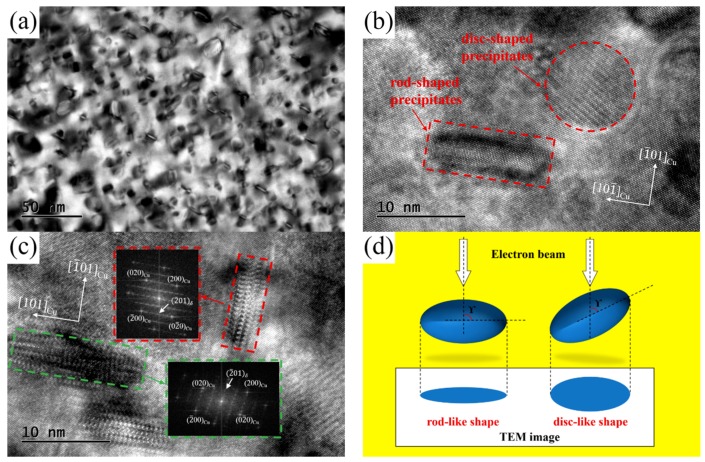
TEM and high resolution transmission electron microscopy (HRTEM) images of the NS-4 alloy aged at 500 °C for 2 h. (**a**) Bright-field TEM image along [110]_Cu_, (**b**) HRTEM images, (**c**) HRTEM images along [001]_Cu_, and (**d**) morphology of the precipitated phase.

TEM and HRTEM images of the NS-4 alloy were obtained to identify the morphology and crystal orientation relationship of precipitates as shown in Figure 9. The different shape characteristic of precipitation particles are observed clearly through the bright field image along the [110]_Cu_ directions as illustrated in Figure 9a. From the HRTEM results in Figure 9b,c, the precipitates of disc and rod shapes are identified in the copper matrix. The relationship between the electron beam and precipitated phase is presented in Figure 9d. The phase shows a rod-like shape when the angle γ of the precipitation phase and incident direction is 90°, otherwise, the phase appears to be disc-like [15]. According to the lattice parameters of the Ni_2_Si phase (a = 0.706 nm, b = 0.499 nm, c = 0.372 nm) and copper matrix (a = b = c = 0.362 nm) [19], the lattice dislocation caused by the Ni_2_Si phase in the direction of axis b and c is smaller than that in the direction of axis a. The growth in the direction of axis b and c is preferred to efficiently minimize the precipitated energy, and the direction of axis a is shortened to present a disc-shaped structure. As discussed above, the true morphological characteristic of these precipitates is a disc-like shape.

**Figure 10:**
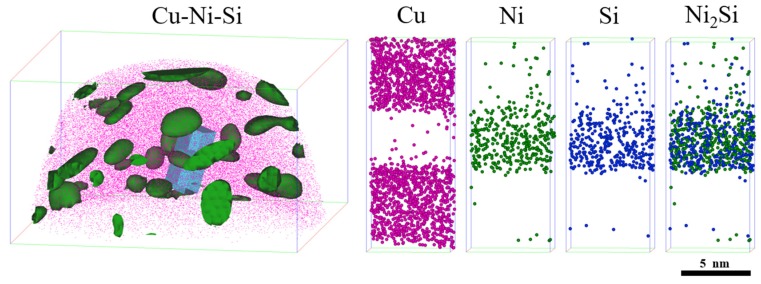
3D atom maps of Cu, Ni, and Si atoms of the NS-4 alloy aged at 500 °C for 2 h.

The 3D approach is adopted for alloy element maps to explore in-depth information on the type and distribution of precipitates in a peak aged NS-4 alloy as shown in Figure 10. A high number density of disc- and rod-like Ni_2_Si precipitates is clearly observed, which is in accordance with the descriptions in Figure 9. Meanwhile, the solute atoms of Ni and Si show strong co-segregation, and the Cu atoms are randomly distributed on the external of these co-segregations in the matrix. These two atoms are uniformly dispersed and alternately distributed within the precipitated phase instead of forming their own atomic cluster structure.

**Figure 11:**
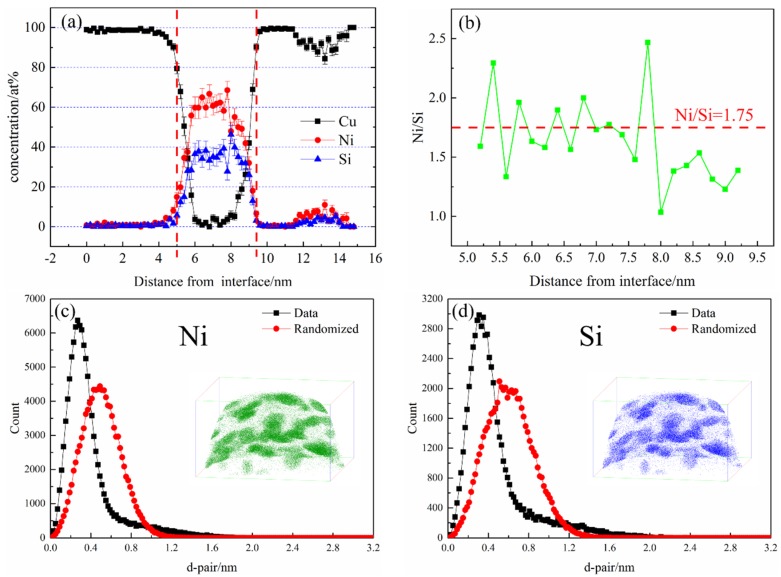
Atomic concentration distribution profiles and nearest neighbor distribution (NND) curves of the NS-4 alloy. (**a**) Atomic concentration distribution profiles, (**b**) enlarged area of the dotted line in (**a**), (**c**) NND curves of Ni and (**d**) NND curves of Si.

Figure 11a shows the atomic concentration distribution profile of the NS-4 alloy by cutting along the perpendicular direction of precipitates in Figure 10. After smoothing, the average concentration of Ni and Si atoms inside the precipitate are approximately 65 at. % and 35 at. %. Figure 11b also shows that the average Ni/Si atomic ratio is 1.75, which is obtained by amplifying the intermediate region of the precipitate in the corresponding atom concentration distribution profile. The atomic configuration is highly consistent with that of the Ni_2_Si phase in the NS-4 alloy. The nearest neighbor distribution (NND) curve represents the difference between the experimental value and standard normal distribution curve [20]. A distinct difference between the two curves indicates serious segregation of solute atoms in the precipitated phase [21]. The NND curves of Ni and Si atoms are presented in Figure 11c,d. The two atoms have strong co-segregation and correspond well with the observations in Figure 10, indicating that the phenomenon occurs in each alloy.

**Figure 12:**
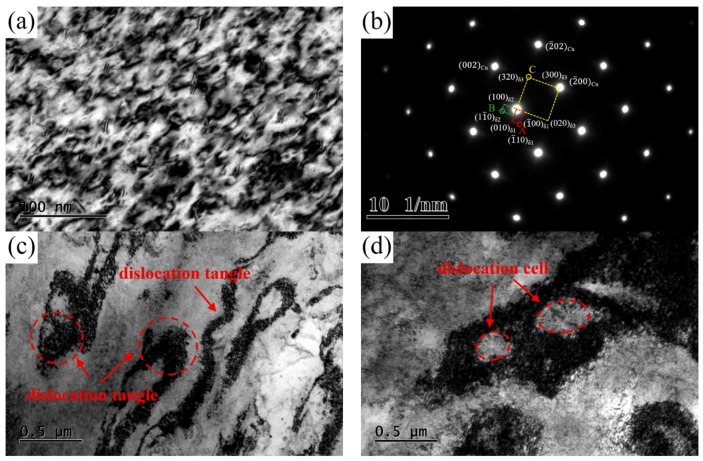
TEM images of the NS-4 alloy with heat treatment of TP-c. (**a**) Bright-field TEM image along [110]_Cu_, (**b**) SAED image corresponding of [001]_Cu_, (**c**) dislocation tangles, and (**d**) dislocation cells.

TEM images of the NS-4 alloy under the peak aging state of TP-c are presented in Figure 12. A large number of dislocations and tangles are observed in the matrix. The type of precipitated phase shows no change according to the SAED pattern image in Figure 12b. Many dislocation tangles and cell structures are captured in the matrix, as shown in Figure 12c,d. These dislocation entanglement regions of dislocation cells increase the storage energy of the alloy, which can be used as the precipitation channel of solute atoms.

**Figure 13:**
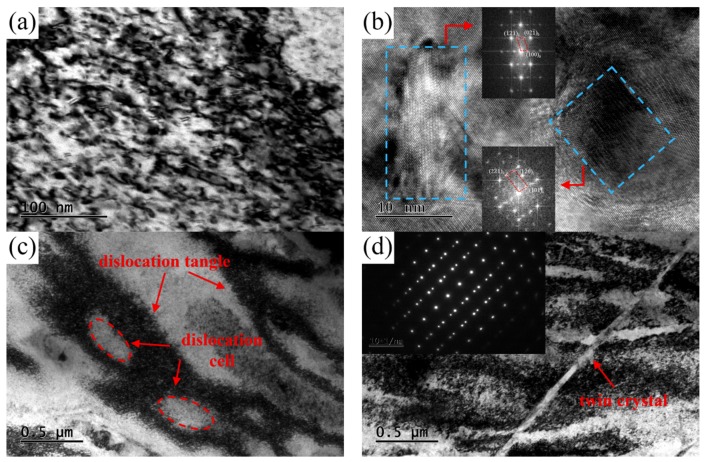
TEM images of the NS-4 alloy with heat treatment of TP-e. (**a**) Bright-field TEM image along [110]_Cu_, (**b**) HRTEM image, (**c**) dislocation tangles and cells, and (**d**) deformation twins and the corresponding SAED image.

**Figure 14:**
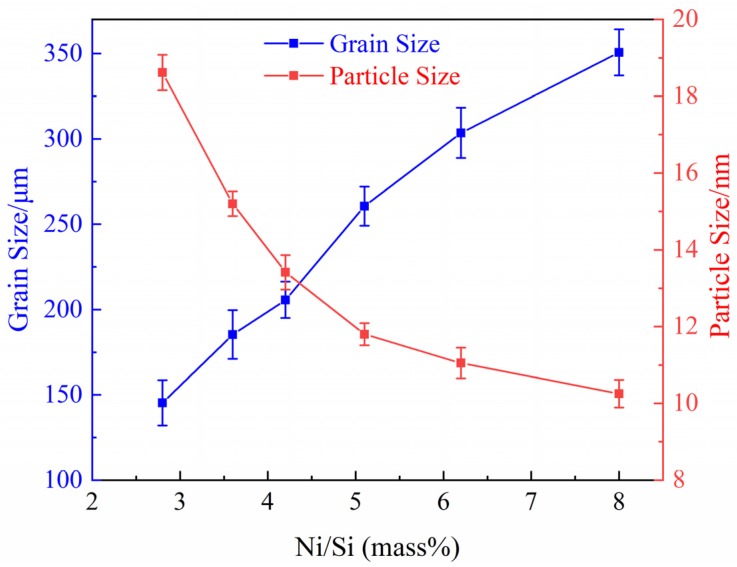
Variation curves of average grain and particle size of alloys with different Ni/Si mass ratios.

Figure 13 shows the TEM images obtained from the NS-4 alloy with combined heat treatment of TP-e. Many dislocation tangles and precipitated particles are also observed in the matrix. Compared with those in the TP-c in Figure 12a, finer precipitated particles and a higher density of dislocations are formed after second cold rolling and aging in Figure 13a. From the HRTEM images in Figure 13b, the precipitates of disc- and rod-like shapes are also identified in the matrix. The characteristic of deformation twin substructure is obtained in Figure 13d, indicating that the severe lattice distortion of alloy occurs during deformation. From the observations in Figure 13 and Figure 14, the growth and nucleation of the precipitated phase are accelerated by these defects, thus enhancing the strength and hardness of the studied alloy.

## 4. Discussion

### 4.1. Effect of Ni/Si Mass Ratio on the Properties of the Cu-Ni-Si Alloy

The strengthening effect for copper alloys can be calculated by linearly accumulating the contributions of the four strengthening mechanisms, namely, solid solution strengthening, grain boundary strengthening, precipitation strengthening, and work hardening [22]. The increase in solid solution strengthening is attributed to the matrix lattice distorted by different sizes of solid solution atoms. The solid solution atoms of Cu-Ni-Si alloy are Ni and Si, and the precipitated phase is mainly the Ni_2_Si phase during the peak aging state. Considering the precipitation of the precipitated phase can remarkably reduce the concentration of atoms in solid solution during subsequent aging. Hence, the difference in the effect of solid solution strengthening on the alloy due to the varying Ni and Si atomic contents can be neglected. Work hardening is primarily caused by cold rolling deformation while the alloy is in the aging state. Hence, the influence of work hardening on the alloy can be ignored. Therefore, the influence of Ni/Si mass ratios on strength is mainly attributed to the precipitation strengthening and grain boundary strengthening.

Precipitation strengthening is the prominent approach for copper alloys to obtain high strength [23]. The movement of dislocations is hindered by the uniform distribution of precipitated particles in the matrix during aging, thus resulting in the strengthening effect. The dominant precipitation strengthening mechanism for Cu-Ni-Si alloy is the Orowan dislocation bypass mechanism, which mainly depends on the average diameter *d_p_* and volume fraction *f_v_* of precipitated phase [24]. The increment of stress Δ*σ_p_* is inversely proportional to the average diameter *d_p_* but proportional to the volume fraction *f_v_*.

(1) ∆σp=0.81MGb2π(1−ϑ)12ln(dp/b)λ−dp

(2) $$\lambda =d_{p}\sqrt{\frac{3\pi }{8f_{v}}}$$

The quantified parameters of precipitates for the alloys with different Ni/Si mass ratios are listed in Table 3. The results reflect the differences in the grain size, particle size, number density, and volume fraction of the alloys. The NS-4 alloy has a significantly higher volume fraction and moderate size of the Ni_2_Si phase than the others, resulting in the better reinforcement of the former. In addition, the NS-4 alloy also has higher quantity density, which indicates its adequate precipitation and further strengthening. According to the calibration and determination of HRTEM and 3DAP above, the type of precipitated phase particles is the Ni_2_Si phase. In theory, the Ni/Si ratio in the atomic fraction of the Ni_2_Si precipitated phase is 2, and the corresponding mass ratio is approximately 4. When the Ni/Si mass ratio is lower or higher than 4, the precipitation kinetics is insufficient because of the excessive Si or Ni atoms, thus reducing the strengthening effect on the alloy. Meanwhile, the purification is inadequate due to the residual Si or Ni particles in the matrix, thereby decreasing the electrical conductivity.

Figure 14 shows the variation of average grain size and particle size of alloys with different Ni/Si mass ratios. The average grain size shows gradual growth with the increase in Ni/Si mass ratios. However, the average size of precipitated phases changes with a contrary tendency. The intersection of two curves appears when the Ni/Si mass ratio is approximately 4 (NS-4), which provides a good combination of the excellent electrical and high mechanical properties of Cu-Ni-Si alloys.

According to the Hall–Petch relationship [25,26,27], *K_y_* is a Hall–Petch coefficient indicating an expression of the effect of surrounding grains on the flow resistance, and *d_g_* is the average diameter of grain size. According to the equation, the square of grain size *d_g_* is inversely proportional to the strength increment *Δσ_GB_*.

(3) $$∆\sigma_{GB}=K_{y}d_{g}^{-1/2}$$

Under the same aging conditions, the growth of grains is inhibited by the precipitated phase particles attached around the grain boundary. Therefore, the large size of precipitated phase particles hinders the growth of grains at the low Ni/Si mass ratios. Strengthening from grain refinement is enhanced owing to the small grains obtained. When the proportion increases, the volume fractions and the number of precipitated phases are reduced, leading to a gradual increase in grain size.

When the Ni/Si mass ratio is approximately 4, the second phases are the most fully precipitated and effectively strengthened throughout the precipitation hardening. Solid solution atoms are dissolved from the supersaturated solid solution, resulting in further purification of the matrix. Meanwhile, strengthening from grain refinement is improved according to the Hall–Petch relationship. The alloy exhibits good electrical conductivity and mechanical properties with the combined strengthening mechanisms.

### 4.2. Effect of Heat Treatment on the Properties of Cu-Ni-Si Alloys

Table 4 displays the comparison of the properties of the NS-4 alloy with different combined treatments under peak aging states. The mechanical properties are remarkably improved without sacrificing electrical conductivity, whereas the elongation is decreased. Another notable phenomenon is that the temperature of peak aging decreases gradually after cold deformation. For example, the peak aging temperature of alloy in the aging state (TP-b) is 500 °C, whereas those of the TP-c and TP-e are 450 °C and 350 °C, respectively.

#### 4.2.1. Precipitate Strengthening

For precipitation strengthening, the increase in yield stress is attributed to the Orowan mechanism based on the interaction between dislocations and Ni_2_Si precipitate particles, which can be expressed by Equations (1) and (2).

Where *M* is the Taylor factor, *G* is the shear modulus of the matrix, *d_p_* is the average diameter of particles, *λ* is the average crystal plane spacing between precipitates, *b* is the Burgers vector, *ν* is the Poisson’s ratio, and *f_v_* is the volume fraction of particles. The relevant parameters and calculated results are summarized in Table 5.

#### 4.2.2. Grain Boundary Strengthening

The boundaries of grain can hinder the movement of dislocations and result in grain boundary strengthening, which can be described by the Hall–Petch relationship and is shown in Equation (3).

Where *K_y_* is Hall-Petch coefficient and *d_g_* is the average diameter of grain size. The relevant parameters and calculated results are summarized in Table 6.

#### 4.2.3. Solid Solution Strengthening

The solid solution strengthening was mainly due to the lattice distortion caused by the solid solution atoms, the increase in yield stress can be expressed by [28]:

(4) $${∆\sigma }_{s}=\sum MG\frac{\varepsilon_{s}^{3/2}c_{x}^{1/2}}{700}$$

(5) $$\varepsilon_{s}=\left|\frac{{\;\;\;\varepsilon \;\;\;}_{G}}{1+\frac{1}{2}\left|{\;\;\;\varepsilon \;\;\;}_{G}\right|}-\beta {\;\;\;\varepsilon \;\;\;}_{b}\right|$$

(6) $$\varepsilon_{G}=\frac{1}{G}\frac{dG}{dc}$$

(7) $$\varepsilon_{b}=\frac{1}{a}\frac{da}{dc}$$

(8) $$c_{x}=\left(1-\eta \right)c_{0}$$

where ε_b_ is the correction factor for the change of lattice parameter; $\varepsilon_{G}$ is the correction factor for the change of the shear modulus, with values that are different due to the different type of solid solution atoms, which is given in Table 7; $c_{x}$ is the atomic concentration of residual solid solution atoms (Ni and Si) in the matrix; $c_{0}$ is the original atomic concentration; η is the precipitating extent of the Ni_2_Si phase; β is a constant 3; and a is the lattice parameter of the copper matrix, which is equal to 0.361 nm. The relevant parameters and calculated results are summarized in Table 8.

#### 4.2.4. Work Hardening

For work hardening, the number and density of dislocations increase during cold rolling, resulting in dislocation strengthening. The increase in yield strength due to dislocations can be estimated by a Taylor relation [29,30,31]:

(9) $$∆\sigma_{d}=M\alpha Gb\rho^{1/2}$$

(10) $$\rho =\frac{2\surd 3\varepsilon }{db}$$

where *M*, *G*, and *b* have the same meaning and values as defined above; α is a constant; and the stress Δ*σ_d_* is proportional to the square root of the dislocation density ρ. ρ can be calculated by Equation (10), where ε is the micro-strain obtained from the XRD analyses of the alloys. The relevant parameters and calculated results are summarized in Table 9.

#### 4.2.5. Calculated Total Strength

**Figure 15:**
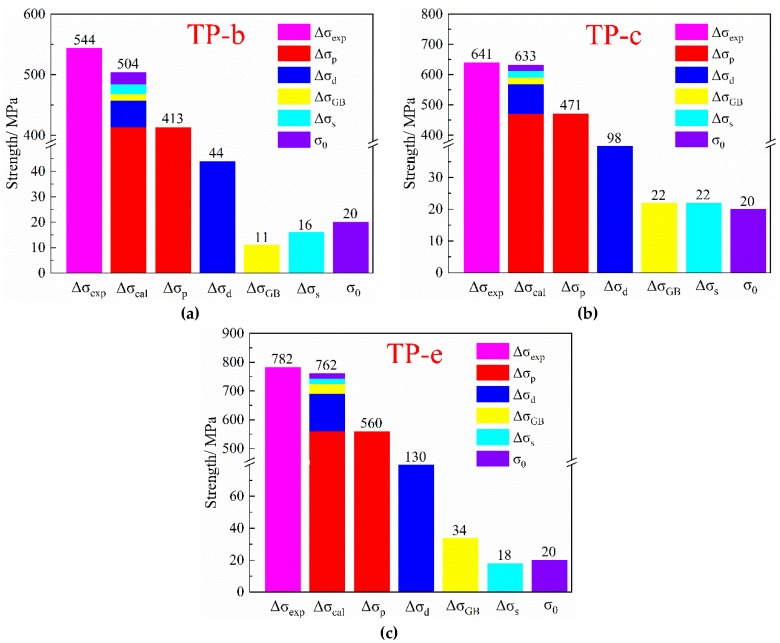
Comparison between experimental and calculated yield strengths with different heat treatments. The combined heat treatments of (**a**) TP-b, (**b**) TP-c and (**c**) TP-e.

The total strength of the alloy is calculated by Equation (11) [22], and the individual contributions of the corresponding mechanisms are listed in Figure 15. The calculated result is highly consistent with the experimental result, and the error values are 7.4%, 1.2% and 2.6%, respectively. The high accuracy of theoretical calculation proves that the work is feasible and applicable in real cases.

(11) $$\sigma_{total}=\sigma_{0}+∆\sigma_{GB}+∆\sigma_{p}+∆\sigma_{s}+∆\sigma_{d}$$

It can be concluded that the effect of different combined heat treatments on strength is mainly attributed to precipitation strengthening and work hardening. As shown in Figure 13 and Table 9, the high density of dislocations is obtained in the TP-e, resulting in a corresponding large strengthening increment Δ*σ_d_*. The dense distribution of dislocations caused by cold rolling and their different strengthening effects on the alloy explain the differences in the hardness curves of TP-b and TP-e [32]. At the same time, the nucleation channels are increased by the dislocation entanglements and deformation twin substructures through the abundant cold rolling, thus accelerating the precipitation of second phases. Subsequently, the lower peak-aging temperature also leads to the lower coarsening rate of precipitates through the multi-stage aging. Therefore, the dense but fine Ni_2_Si precipitates are formed and result in the greater precipitation strengthening increment Δ*σ_p_*.

#### 4.2.6. Conductive Mechanism

The conductive mechanism for Cu-Ni-Si alloys can be calculated by linearly accumulating the contributions of the four corresponding conductive mechanisms, which can be expressed by the Mattiessen rule [28]:

(12) $$\rho_{total}=\rho_{0}+∆\rho_{GB}+∆\rho_{p}+∆\rho_{s}+∆\rho_{d}$$

where $\rho_{0}$ is the electrical resistivity of the copper matrix; and $∆\rho_{GB},\;∆\rho_{p},∆\rho_{s}$ and $\;∆\rho_{d}$ are the electrical resistivity increments caused by the grain boundary, precipitation, solid solution atoms, and dislocation, respectively [33]. The average grain size is coarse and the dislocation density is moderately low in the alloy, so the effects of these factors can be ignored. Furthermore, the precipitate phase in the alloy has little effect on electrical conductivity. Therefore, ∆*ρ_s_* is the most important factor affecting electrical conductivity.

Based on the results shown in Table 4, the studied alloy keeps a good electrical conductivity after proper heat treatment processes. This finding contributes to the great precipitation kinetics of the alloy through the abundant cold deformation. The precipitating extent of the second phase is sufficient, and the contents of Ni and Si atoms in the matrix are substantially reduced to form Ni_2_Si precipitates. Thus, strain energy is effectively reduced, and the matrix is further purified, resulting in the remarkable improvement in mechanical properties without sacrificing electrical conductivity.

### 4.3. Property Comparison

**Figure 16:**
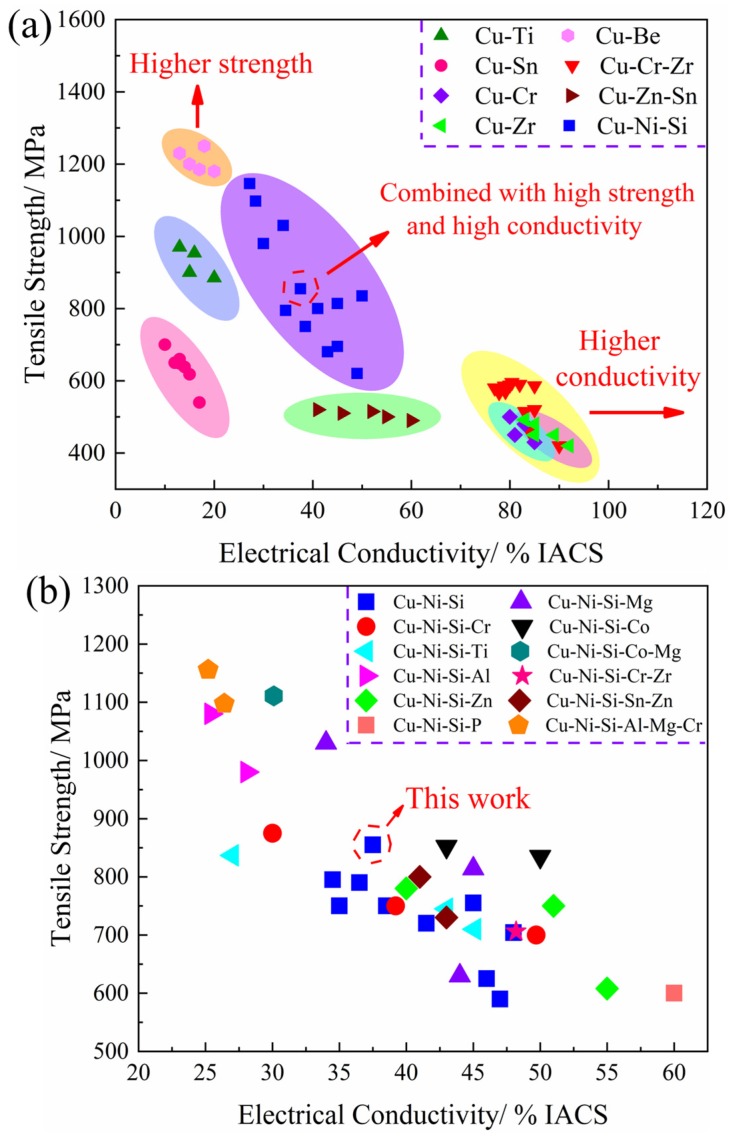
Comparison of tensile strength and electrical conductivity of high-strength copper alloys. (**a**) Cu-Ni-Si systems alloys and other copper alloys [34,35,36,37,38,39,40,41,42,43,44,45,46,47,48,49,50,51] and (**b**) the designed alloy and other Cu-Ni-Si-X alloys [1,2,3,4,52,53,54,55,56,57,58,59].

Figure 16 shows the comparison of tensile strength and electrical conductivity of Cu-Ni-Si system alloys and other kinds of copper alloys such as Cu-Be [34,35,36], Cu-Ti [37,38,39,40], Cu-Sn [41,42], Cu-Cr [24,43,44], Cu-Zr [45,46,47], Cu-Zn-Sn [48,49], and Cu-Cr-Zr [50,51]. The current development trend of copper alloys is mainly divided in three directions. The first type of alloys with high strength is the representative Cu-Be alloy, which exhibits superior hardness and strength (>1100 MPa). However, its low conductivity (>20% IACS) and high stress relaxation properties are also evident. Most importantly, the toxicity of beryllium is harmful to physical health, further restricting its development and application. The second type of alloys are dominated by those with high conductivity, mainly including Cu-Cr, Cu-Zr, and Cu-Cr-Zr alloys. These kinds of alloys are known for their excellent electrical (>70% IACS) and thermal conductivity and good anti-softening performance at elevated temperatures. Their feature of low strength (<600 MPa) and difficult preparation greatly affects the practicability of these alloys. For example, smelting of the Zr element must be conducted in a non-vacuum environment, which requires an extremely strict environment and greatly increases the production costs. The third type shows the properties of high strength and conductivity as represented by the Cu-Ni-Si system alloys. As displayed in Figure 16a, the Cu-Ni-Si system alloys exhibit outstanding combination properties with electivity conductivity of 30–50% IACS and tensile strength of 600–1000 MPa, making them one of the main research hotspots in the field of elastic materials.

Figure 16b shows the comparison results of Cu-Ni-Si system alloys by adding different alloy elements. The properties of alloys greatly vary with the addition of different alloy elements. For instance, the multi-element alloy of Cu-Ni-Si-Al-Mg-Cr [4,28] has the highest tensile strength, reaching 1100 MPa with a corresponding conductivity of 25% IACS. The highest conductivity is obtained from the quaternary alloy of Cu-Ni-Si-P [58] with properties of 60% IACS and 600 MPa. Compared with the ternary Cu-Ni-Si alloy, the alloy obtained in the present study exhibits improved strength and comprehensive performance. Therefore, this kind of alloy can be used as the basis of a new alloy design, in which trace alloying elements are added to further enhance its electrical conductivity and strength. Studies on the different Ni/Si mass ratios and combined treatments for the ternary alloy also provide a foundation for the design of a new multicomponent alloy with ultrahigh strength and excellent electrical conductivity.

## 5. Conclusions

In this article, the properties and microstructure evolution of ternary Cu-Ni-Si alloys with different Ni/Si mass ratios are discussed systematically. The Cu-Ni-Si alloy with Ni/Si mass ratio of 4–5 shows an excellent combination of strength, hardness, and electrical conductivity. These precipitates of disc-like shape are confirmed as Ni_2_Si phase with orthorhombic structure based on the TEM, HRTEM, and 3DAP characterization. After being hot rolled by 85% at 930 °C, the solution was treated at 900 °C for 1 h. First cold roll by 60% and first aging at 450 °C for 1 h were conducted, followed by second cold roll by 45% and second aging at 350 °C for 1 h. The studied alloy exhibits the following excellent properties: micro-hardness of 290 HV, electrical conductivity of 37.5% IACS, tensile strength of 855 MPa, yield strength of 782 MPa, and elongation percentage of 4.5%. The high strength was primarily attributed to the Orowan precipitation strengthening and secondarily attributed to the work hardening. Quantitative analyses between the heat treatments and properties were conducted and were found to coincide well with the experimental results.

## Figures and Tables

**Table 1:** Tested chemical compositions of Cu-Ni-Si alloys, wt %.

Alloy	Ni	Si	Ni/Si
NS-1	3.20–3.40	1.10–1.17	2.8–3.0
NS-2	3.20–3.40	0.95–1.00	3.3–3.5
NS-3	3.20–3.40	0.83–0.90	3.6–4.0
NS-4	3.20–3.40	0.73–0.80	4.2–4.5
NS-5	3.20–3.40	0.60–0.65	5.1–5.5
NS-6	3.20–3.40	0.54–0.55	6.0–6.2
NS-7	3.20–3.40	0.47–0.50	6.6–7.0
NS-8	3.20–3.40	0.44–0.45	7.3–7.5
NS-9	3.20–3.40	0.40–0.41	8.0–8.3

**Table 2:** Combined heat treatments for the NS-4 alloy.

No.	Combined Heat Treatment Processes
TP-a	Solution treated + aged at 450 °C for different times
TP-b	Solution treated + aged at 500 °C for different times
TP-c	Solution treated + cold rolled by 60% + aged at 450 °C for different times
TP-d	Solution treated + cold rolled by 60% + aged at 500 °C for different times
TP-e	Solution treated + cold rolled by 60% + first aged at 450 °C/1 h + cold rolled by 45% + second aged at 350 °C for different times

**Table 3:** Quantitative measurements of NS-1, NS-4, and NS-9 alloys.

Alloy	Grain Size/μm	Particle Size/nm	Number Density /(10^22^/m^3^)	Volume Fraction/%
NS-1	145 ± 13.32	18.62 ± 0.49	3.47 ± 0.51	2.65 ± 0.43
NS-4	205 ± 10.61	13.41 ± 0.45	8.21 ± 0.37	4.96 ± 0.22
NS-9	350 ± 13.58	10.25 ± 0.36	6.43 ± 0.24	3.48 ± 0.16

**Table 4:** Mechanical properties and electrical conductivity of the NS-4 alloy with different combined heat treatments.

Conditions Properties	TP-b	TP-c	TP-e
Peak aging process	ST + 500 °C /2 h	ST + 60% CR + 450 °C /1 h	ST + 60% CR + 450 °C/1 h + 45% CR + 350 °C/1 h
Hardness (HV)	238 ± 5.08	260 ± 6.67	290 ± 7.15
Conductivity (% IACS)	37.5 ± 1.05	37.6 ± 1.18	37.5 ± 0.69
Tensile strength (MPa)	651 ± 18.74	715 ± 23.13	855 ± 15.26
Yield strength (MPa)	544 ± 12.53	641 ± 19.75	782 ± 17.11
Elongation (%)	14.3 ± 0.68	8.0 ± 0.52	4.5 ± 0.44

**Table 5:** Relevant parameters and calculated results of the precipitation strengthening.

Heat Treatment	$d_{p}/nm$	$f_{v}/\%$	*M*	*G*/GPa	*b*/nm	ϑ	$∆\sigma_{P}/\mathrm{MPa}$
TP-b	13	4.96	3.06	44	0.255	0.3	413
TP-c	11	5.03	3.06	44	0.255	0.3	471
TP-e	9	5.24	3.06	44	0.255	0.3	560

**Table 6:** Relevant parameters and calculated results of the grain boundary strengthening.

Heat Treatment	$d_{g}/\mu m$	$K/\mathrm{MPa}\cdot \mu m^{1/2}$	$∆\sigma_{g}/\mathrm{MPa}$
TP-b	200	150	11
TP-c	45	150	22
TP-e	20	150	34

**Table 7:** Relevant parameters of solid solution atoms in alloys.

Solute Atoms	Lattice Parameter/nm	Shear Modulus/GPa	$\varepsilon_{b}$	$\varepsilon_{G}$	$\varepsilon_{s}$
Ni	0.352	77	−0.025	0.674	0.559
Si	0.543	65	0.504	0.413	1.17

**Table 8:** Relevant parameters and calculated results of the solid solution strengthening.

Heat Treatment	η/%	$c_{Ni}/\mathrm{at}.\%$	$c_{Si}/\mathrm{at}.\%$	$∆\sigma_{Ni}$	$∆\sigma_{si}$	$∆\sigma_{s}/\mathrm{MPa}$
TP-b	88	0.43	0.22	5	11	16
TP-c	78	0.79	0.39	7	15	22
TP-e	87	0.47	0.23	6	12	18

**Table 9:** Relevant parameters and calculated results of the work hardening.

Heat Treatment	$\rho /m^{-2}$	ε	*M*	*G*/GPa	*b*/nm	α	$∆\sigma_{d}/\mathrm{MPa}$
TP-b	4.15 × 10^13^	0.61	3.06	44	0.255	0.2	44
TP-c	2.03 × 10^14^	0.67	3.06	44	0.255	0.2	98
TP-e	3.56 × 10^14^	0.52	3.06	44	0.255	0.2	130
